# Effects of cigarette smoking on retinal thickness and choroidal vascularity index: a systematic review and meta-analysis

**DOI:** 10.1186/s40942-025-00646-9

**Published:** 2025-02-26

**Authors:** Miguel A. Quiroz-Reyes, Erick A. Quiroz-Gonzalez, Miguel A. Quiroz-Gonzalez, Virgilio Lima-Gomez

**Affiliations:** 1 The Retina Department of Oftalmologia Integral ABC Medical and Surgical Nonprofit Organization, National Autonomous University of Mexico, Lomas de Chapultepec, Av. Paseo de las Palmas 735 Suite 303, Mexico City, 11000 Mexico; 2 Juarez Hospital, Public Assistance Institution (Nonprofit Organization), Av. Politecnico Nacional 5160, Colonia Magdalena de las Salinas, Mexico City, 07760 Mexico

**Keywords:** Choroidal thickness, Choroidal vascularity index, Full-retinal thickness, Cigarette smoking, Optical coherence tomography angiography, Ocular health, Oxidative stress, Subfoveal choroidal thickness

## Abstract

**Background:**

Smoking increases oxidative stress, affecting the vascular endothelium by decreasing the antioxidant vitamin C and disrupting regular nitric oxide activity. It reduces blood flow in the retina and choroid due to increased vascular resistance and compromised choroidal blood flow regulation compared to nonsmokers. This systematic review and meta-analysis aimed to elucidate the impact of cigarette smoking on retinal thickness and the choroidal vascularity index (CVI).

**Methods:**

A comprehensive literature search was performed across multiple databases, including Web of Science, Medline, PubMed, and Embase, adhering to the PRISMA and MOOSE guidelines. Observational studies were selected to explore the relationships between smoking and ocular parameters such as CVI, full-retinal, and choroidal thickness. Two independent reviewers conducted the data extraction and quality assessment using a modified Newcastle–Ottawa scale. Statistical analysis was performed using a random-effects model.

**Results:**

Four out of the 743 identified articles, involving 702 eyes, met the inclusion criteria. The analysis revealed a significant reduction in the CVI among smokers (SMD: -0.61, 95% CI: -0.78 to -0.43, *p* < 0.00001), indicating compromised choroidal vascularity. In contrast, the impact of smoking on subfoveal choroidal thickness (SFCT) was not statistically significant (mean difference: 3.88 μm, 95% CI: -7.34 to 15.10, *p* = 0.50), with high heterogeneity (I² = 79%). Additionally, the full-retinal thickness (FRT) did not show a significant difference between smokers and nonsmokers.

**Conclusion:**

Cigarette smoking negatively affects choroidal vascularity, as indicated by a significant reduction in CVI. However, its impact on FRT and SFCT remains unclear and requires further research. These findings highlight the importance of smoking cessation for eye health and suggest that CVI is a valuable noninvasive biomarker for monitoring vascular changes in smokers.

**Trial registration:**

PROSPERO registration number: CRD42024627478.

**Supplementary Information:**

The online version contains supplementary material available at 10.1186/s40942-025-00646-9.

## Background

Cigarette smoking, a highly adaptable environmental factor, is an independent risk factor for many systemic diseases, with projections indicating that approximately 9 million people will die from smoking annually by 2030 [1–3]. Cigarette smoking is strongly associated with cardiovascular diseases, including myocardial infarction and atherosclerosis [4–7]. Ocular healthcare professionals emphasize a significant correlation between smoking and common ocular-threatening conditions, including age-related macular degeneration (AMD), diabetic retinopathy, cataracts, contact lens-related keratitis, and Graves’ ophthalmopathy [8, 9]. It has also been reported to contribute to various fundus diseases, including AMD, ischemic optic neuropathy, and glaucoma [10–12]. Although the specific pathophysiological mechanism involved remains unclear, endothelial dysfunction is believed to play a pivotal role in the development of smoking-induced vascular diseases, according to Wimpissinger et al. [13]. Smoking increases oxidative stress, influencing the vascular endothelium by decreasing the antioxidant vitamin C and disrupting regular nitric oxide activity. In addition, smoking reduces blood flow in the retina, secondary to increased vascular resistance and impaired regulation of choroidal blood flow compared to nonsmokers [13].

Numerous researchers have investigated choroidal tissue in vivo using advanced optical coherence tomography (OCT) in a non-invasive manner [13–15]. OCT angiography (OCTA), a novel noninvasive imaging strategy, has recently been introduced for vascular mapping without dye injection. OCTA provides high-speed, 3D images of the retinal, choroidal, and optic disc vasculature [16]. Studies have shown normal vascular mapping of the healthy retina, an age-related reduction in retinal capillary density at both superficial and deep plexuses, and an age-related increase in the foveal avascular zone [17–19]. Choroidal thickness (CT) has been assessed primarily as a parameter representing the choroid’s state, and smoking’s effect on CT has been investigated in multiple studies [13–15, 20–24]. CT was initially used as a surrogate marker to evaluate the choroid. However, it has now largely been replaced by the choroidal vascularity index (CVI), which provides a thorough analysis of the choroidal vascular system using Enhanced Depth Imaging (EDI) with A-scan OCT. Unlike OCTA, which maps retinal and choroidal vasculature, CVI specifically quantifies the choroid’s stromal and luminal components. According to Wei et al. [15], healthy smokers exhibited a significantly lower CVI compared to healthy controls. This reduction in CVI among smokers may be attributed to a decreased luminal area (LA), an increased stromal area (SA), or a combination of both, likely due to chronic smoking-related vascular dysfunction and inflammation. The exact underlying mechanism was not examined in this study; however, a plausible hypothesis can be drawn from the known effects of cigarette smoking on blood vessels in other parts of the body. Reduced LA could result from impaired vasodilation due to endothelial dysfunction, while increased SA might stem from a chronic proinflammatory response leading to exudation and fibrosis. The study highlighted its potential as a valuable marker for assessing the effects of smoking on choroidal health.

This systematic review and meta-analysis aimed to examine how cigarette smoking impacts CVI, clarifying the complex relationship between smoking and ocular microvascular dynamics. The study offers critical insights into retinal health by evaluating the superficial and deep capillary plexuses and vessel density (VD) in macular OCTA slabs. Key indicators of choroidal perfusion, such as CVI and choriocapillaris flow area (CFA), are essential for assessing choroidal blood flow. Additionally, the study explores the relationship between visual acuity and choroidal perfusion indices, such as CVI, especially in individuals with a history of chronic cigarette smoking.

## Methodology

### Search strategy

This systematic review and meta-analysis was conducted according to the Preferred Reporting Items for Systematic Reviews and Meta-Analyses (PRISMA) [27] guidelines and the Meta-analysis of Observational Studies in the Epidemiology Group (MOOSE) [28]. It was registered with Prospero under registration number CRD42024627478, which can be consulted at the following link: https://www.crd.york.ac.uk/prospero/display_record.php?RecordID=627478. Two reviewers (MAQR and EAQG) conducted a literature search on the Web of Science, Medline, PubMed, and Embase databases. The detailed search strategy is listed in the Supplementary information file. The following keywords were used for relevant study identification: “cigarette,” “smoking,” “nicotine,” and “tobacco,” in combination with “choroidal vascularity index,” “CVI,” “full-retinal thickness,” “retinal nerve fiber layers,” and “choroidal thickness.”

In addition, we reviewed the reference lists of the eligible studies for additional publications. This systematic review and meta-analysis included observational studies, encompassing both cross-sectional and case-control investigations that examined the impact of smoking on changes in the CVI. The literature search was restricted to English only.

### Study selection criteria

Two reviewers (MAQR and EAQG) independently evaluated all studies retrieved from the databases. Studies were identified as potentially eligible based on their titles and abstracts. These potentially eligible studies underwent further full-text screening to select those that were fully aligned with our objectives. The patients met the eligibility criteria for the systematic review and meta-analysis. The inclusion criteria for studies to be considered eligible were as follows: (a) studies with observational designs, including cross-sectional, case-control, or cohort studies, comparative studies, and randomized controlled trials (RCTs); (b) studies explicitly examining the effects of cigarette smoking on retinal and choroidal thickness or the CVI, providing detailed data for these parameters. The exclusion criteria for this study included non-human research, studies that did not report data on CVI or retinal and choroidal thickness, and studies that lacked information on smoking status or failed to report associations between smoking and ocular parameters. Two reviewers (MAQG and VLG) independently selected the studies. Any disagreements were resolved through discussion between the reviewers or with a conciliator (MAQG).

### Data extraction

Two reviewers (MAQR and EAQG) independently extracted data from eligible studies, following the predetermined inclusion criteria. The data were recorded based on the following information: the first author’s name, study location, participant demographics (quantity, sex, age), methods used for detecting retina/choroid thickness, smoking definitions, and whether adjustments were made for confounding factors. Mean values with standard deviations (SDs) were extracted and calculated.

### Quality assessment

A modified scoring scale based on the Newcastle–Ottawa Scale (NOS) for observational studies, as developed by Yang et al. (2019) [29] was utilized. The rating system established by Yang et al. (2019) [29] assigned one point for each of the following criteria: (1) adoption of a prospective study design; (2) utilization of a case-control study design; (3) presentation of appropriate inclusion and exclusion criteria for all participants; (4) matching of general characteristics, such as age and sex, across the participant population; (5) matching of other potential confounding factors, such as refractive error and axial length; (6) establishment of a reasonable definition for the smoking group; (7) provision of detailed information regarding the control group; (8) acquisition of OCT data for retina/choroid thickness measurement; and (9) description of the detailed detection method and progression. A higher total score indicated a higher study quality. For this investigation, studies with a quality score exceeding six points were classified as high quality.

### Meta-analysis

The impact of cigarette smoking on the retina or CT was investigated through a meta-analysis by pooling data from the included studies. The data are presented as pooled standardized mean differences (SMDs) with 95% confidence intervals (CIs) in forest plots. Heterogeneity was assessed using the *χ*^2^ test and *I*^2^ test, with a p value set at *P* < 0.10 or *I*^2^ > 50%. Because observational studies were exclusively included, a random-effects model was applied. Subgroup analysis stratified by study design, anatomical structure, and detection equipment was conducted to explore potential sources of heterogeneity. The sensitivity analysis involved removing one study at a time and excluding studies with quality scores of less than 5 points. A leave-one-out additional sensitivity analysis on cigarette smoking and CVI was conducted to assess the robustness of the pooled effect size. Begg’s and Egger’s tests were employed to detect publication bias and recognize the limitations of the funnel plots. Statistical analyses were conducted using R programming (version 4.3.1), with a significance level of *P* < 0.05 unless otherwise specified.

### Dose-response analysis between smoking intensity and choroidal vascularity index

To explore the dose-response relationship between cigarette smoking intensity and CVI, we conducted a regression analysis using pack-year data as an independent variable and CVI as the dependent variable. The “pack-year” is a standard measure of cumulative smoking exposure, calculated by multiplying the number of packs of cigarettes smoked per day by the number of years a person has smoked. Pack-year data were available from Wei et al. (2019) [15], Eski et al. (2023) [30], Koçak et al. (2020) [31], and Okawa et al. (2021) [32]. CVI measurements from smokers and nonsmokers were extracted from these studies. Only studies that reported both pack years and CVI were included in the dose-response analysis.

The dependent variable was the CVI (%), and the independent variable was each study’s mean pack-year value (Mean ± SD). Pearson’s correlation coefficient (r) was used to evaluate the strength and direction of the association between smoking intensity and CVI. A P-value less than 0.05 was deemed statistically significant.

## Results

### Characteristics of the retrieved studies

**Fig. 1:**
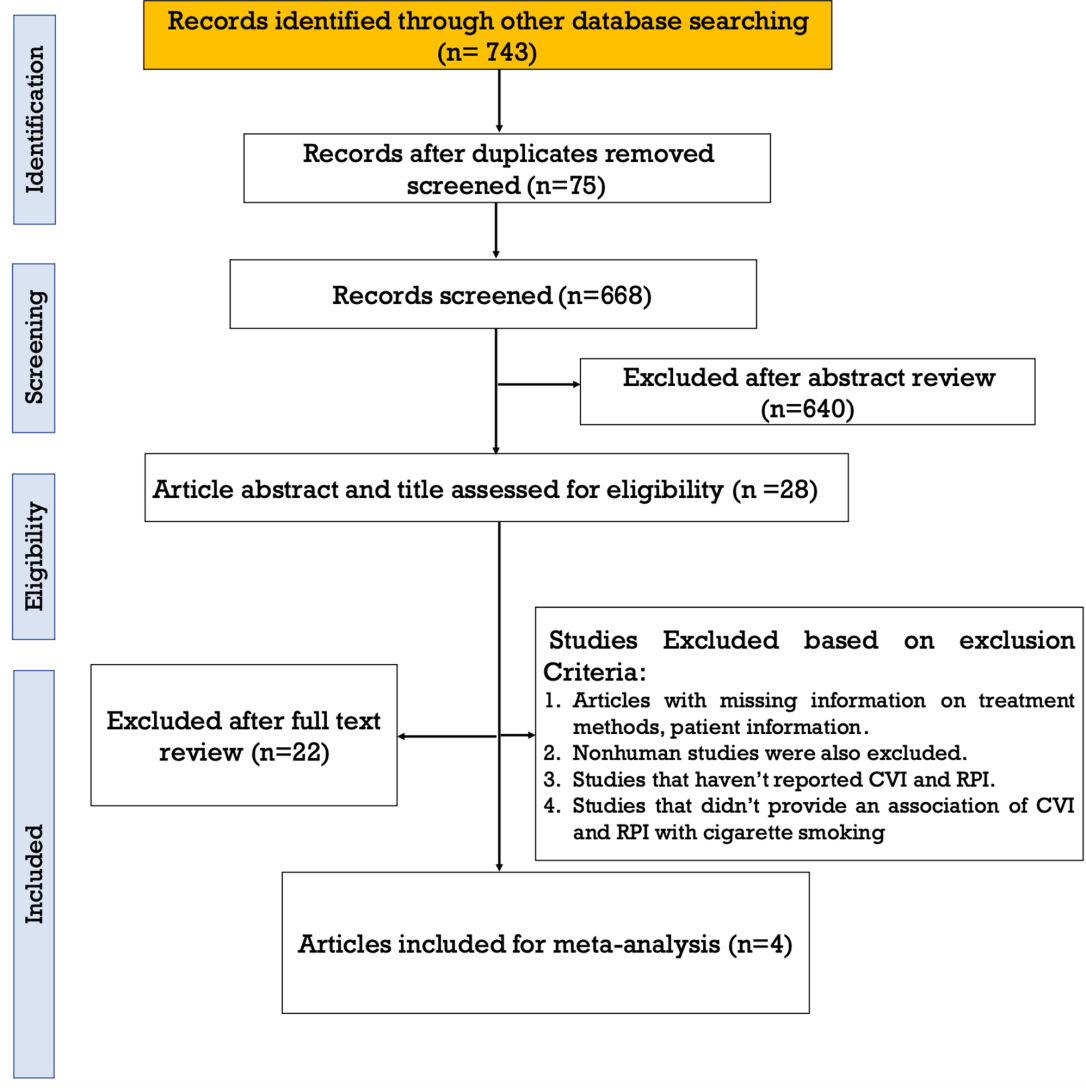
PRISMA flow diagram of all the retrieved articles included in this meta-analysis

A total of 743 articles were identified through the initial database search. Initially, 75 duplicate records were removed, leaving 668 studies for screening. During the screening phase, 640 documents were excluded, including reviews, case reports, correspondences, abstracts, and irrelevant studies. An additional 20 studies were excluded based on their titles and abstracts. Two remaining studies were excluded due to insufficient data and irrelevant interventions. The full-text assessment revealed that four studies in English met the criteria for inclusion in the meta-analysis (Fig. 1). All selected studies were comparative, nonrandomized, retrospective, and prospective. No other novel comparative interventions were identified in this study. A total of 702 eyes were included in the four included studies. The detailed characteristics of the studies are presented in Table 1.

### Impact of cigarette smoking on the choroidal vascularity index

**Fig. 2:**
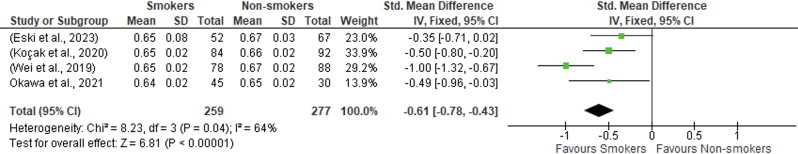
Forest plot illustrating the impact of cigarette smoking on the CVI

The meta-analysis of the four included studies comparing the CVI between smokers and nonsmokers revealed a significant (*P* < 0.00001) overall effect favoring nonsmokers (Fig. 2). The SMD was − 0.61, with a 95% CI ranging from − 0.78 to -0.43 (*P* < 0.00001), indicating that nonsmokers had a greater CVI than smokers. Each study consistently showed a negative SMD, with individual SMDs ranging from − 1.00 (Wei et al.., 2019) [15] to -0.35 (Eski et al.., 2023) [30]. The heterogeneity among the studies was moderate (I² = 64%), suggesting some variation in the effect sizes. These findings indicate that smoking is significantly associated with a lower CVI.

### Impact of cigarette smoking on subfoveal choroidal thickness

**Fig. 3:**
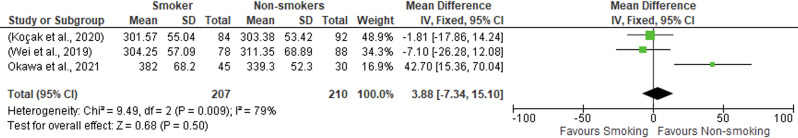
Forest plot illustrating the impact of smoking on SFCT

We evaluated the impact of smoking on subfoveal choroidal thickness (SFCT) (Fig. 3), and three studies were included in this analysis: Koçak et al. (2020) [31], Wei et al.. (2019) [15], and Okawa et al.. (2021) [32]. Koçak et al.. (2020) [31] reported a mean SFCT of 301.57 μm (SD 55.04) in smokers and 303.38 μm (SD 53.42) in nonsmokers, resulting in a mean difference of -1.81 μm (95% CI: -17.86 to 14.24), accounting for 48.9% of the overall weight. Wei et al.. (2019) [15] reported a mean SFCT of 304.25 μm (SD 57.09) in smokers compared to 311.35 μm (SD 68.89) in nonsmokers, resulting in a mean difference of -7.10 μm (95% CI: -26.28 to 12.08), with a weight of 34.3%. In contrast, Okawa et al.. (2021) [32] reported a significantly greater SFCT in smokers (mean 382 μm, SD 68.2) compared to nonsmokers (mean 339.3 μm, SD 52.3), showing a mean difference of 42.70 μm (95% CI: 15.36 to 70.04), representing 16.9% of the overall weight. The combined analysis, incorporating data from 207 smokers and 210 nonsmokers, yielded an overall mean difference of 3.88 (95% CI: -7.34 to 15.10), as shown in Fig. 3. Despite the individual variances observed across the studies, the overall effect was not statistically significant (Z = 0.68, *P* = 0.50). However, considerable heterogeneity among the studies (χ² = 9.49, df = 2, *P* = 0.009; I² = 79%) indicated variability in the effect estimates. This heterogeneity suggests that factors other than smoking status may influence SFCT, warranting further investigations into potential confounding variables and study-specific characteristics.

### Impact of cigarette smoking on full-retinal thickness

**Fig. 4:**
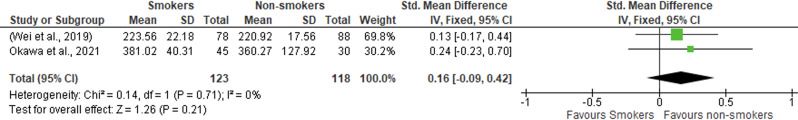
Forest plot illustrating the impact of smoking on FRT

A meta-analysis was conducted to compare the full-retinal thickness (FRT) outcomes between smokers and nonsmokers across two studies The SMD for Wei et al. (2019) [15] was 0.13 (95% CI: -0.17, 0.44), and that for Okawa et al. (2021) [32] was 0.24 (95% CI: -0.23, 0.70), as shown in Fig. 4. The overall pooled SMD was 0.16 (95% CI: -0.09, 0.42), indicating a small positive effect favoring smokers, although this difference was not statistically significant. Heterogeneity was low (Chi² = 0.14, df = 1, *P* = 0.71; I² = 0%), suggesting study consistency. The test for the overall effect yielded a Z value of 1.26 (*P* = 0.21), supporting the lack of a significant difference between smokers and nonsmokers.

In contrast to the frequently reported CVI, SFCT, and FRT, the retinal perfusion index (RPI) has not yet been reported in the literature. This underscores a gap in research concerning the vascular effects of smoking on retina health.

### Quality assessment and publication bias

The results of the quality assessment of the included nonrandomized studies are presented in Table 2. Each study was evaluated using a series of methodological items, with scores ranging from 0 to 2 for each item. The methodological index for nonrandomized studies (MINORS) scores ranged from 17 to 23, indicating varying levels of methodological quality. Koçak et al. (2020) [31] achieved the highest scores (23), suggesting this was the most robust methodology among the included studies. In contrast, Eski et al. (2023) [30] reported the lowest scores (17), indicating potential areas for improvement in study design and reporting. Overall, the quality assessment highlights the variability in methodological rigor across the studies, which should be considered when interpreting the meta-analysis results.

**Fig. 5:**
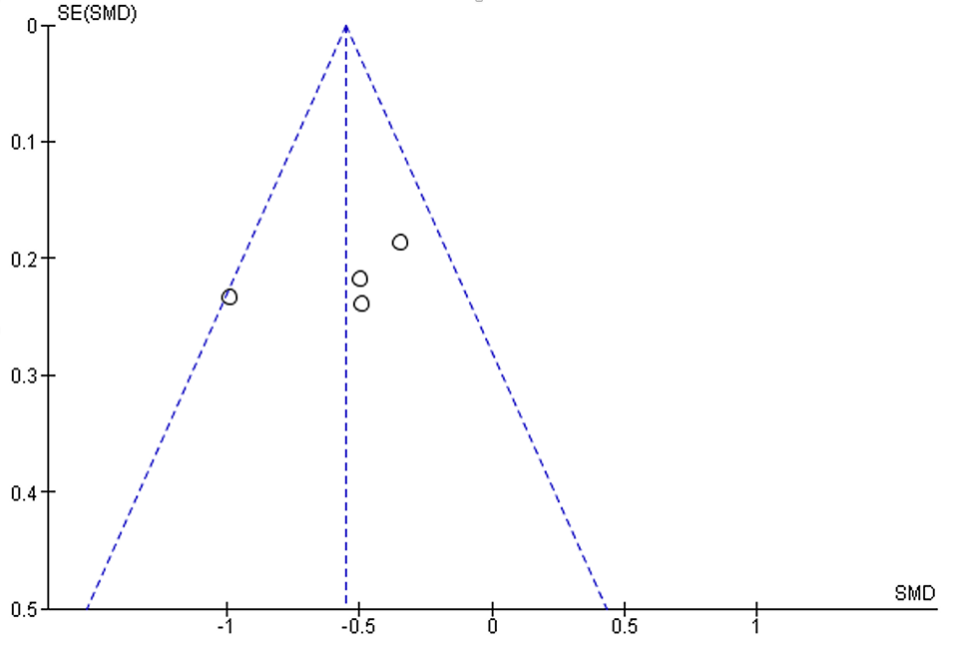
Funnel plot evaluating potential publication bias in the standardized mean difference (SMD) meta-analysis

We generated a funnel plot (Fig. 5) to assess the potential for publication bias. The plot displays the SMDs from the included studies against their standard errors (SEs). The plot appeared symmetrical, with the data points distributed evenly around the central line, indicating a low risk of publication bias. The studies fell within the expected range, with no evident gaps or asymmetry, suggesting the preferential publication of studies with significant results. Thus, the meta-analysis results are unlikely to be significantly influenced by publication bias.

### Sensitivity analysis (leave-one-out method)

**Fig. 6:**
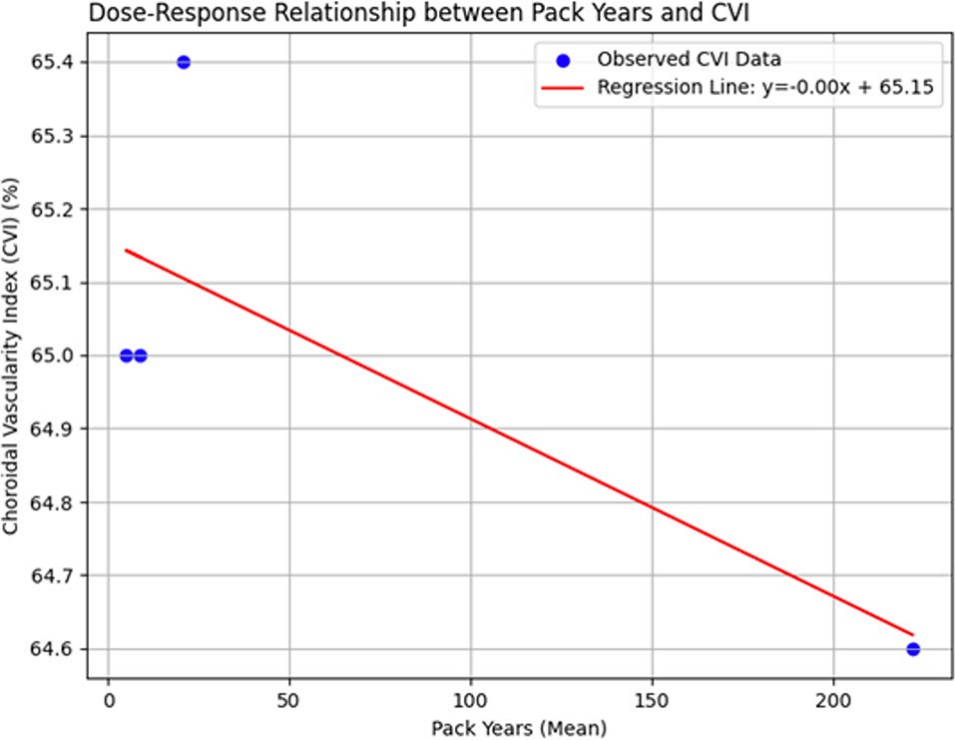
Leave-One-Out Sensitivity Analysis on Cigarette Smoking and CVI. After sequentially removing each study, the plot displays the pooled effect size (SMD) and the 95% confidence intervals, indicating no influence on the meta-analysis results

A leave-one-out sensitivity analysis was conducted to assess the relationship between smoking and CVI, aiming to evaluate the strength and improve the reliability of the pooled effect size. The overall effect remained statistically significant (Fig. 6) across all exclusions, confirming that no study disproportionately influenced the meta-analysis results. The most notable change occurred when Wei et al. (2019) [15] was removed, increasing the pooled effect size to -0.45 (95% CI: -0.65, -0.24), indicating that this study contributed to heterogeneity. Nevertheless, the direction of the effect remained consistent throughout all analyses, highlighting the robustness of the observed association between cigarette smoking and decreased CVI.

**Table 1:** Study characteristics of the included studies

Author’s	Study Design	Smoking Status	Number of participants (No. (%))	Gender (Male/Female)	Number of eyes (No. (%))	Mean age (years, mean ± SD)	CVI (Mean ± SD) %	FRT (µm, Mean ± SD)	SFCT (µm, Mean ± SD)	Pack year (Mean ± SD)
Wei et al. (2019) [15]	Cross sectional observational study	Smokers	39 (46.9)	39/-	78 (46.9)	41.79 ± 6.48	0.65 ± 0.02	223.56 ± 22.18	304.25 ± 57.09	8.89 ± 5.33
		Nonsmokers	44 (53.1)	44/-	88 (53.1)	36.56 ± 8.77	0.67 ± 0.02	220.92 ± 17.56	311.35 ± 68.89	-
Eski et al. (2023) [30]	Retrospective	Smokers	52	-	52 (43.7)	23.76 ± 1.84	0.65 ± 0.08	-	-	*5 ± 2.75*
		Nonsmokers	67	-	67 (56.3)	23.98 ± 2.18	0.67 ± 0.03	-	-	-
Koçak et al. (2020) [31]	Prospective	Smokers	42	42/-	84	43.1 ± 7.26	0.654 ± 0.023		301.57 ± 55.04	20.63 ± 6.53
		Nonsmokers	46	46/-	92	41.82 ± 9.92	0.663 ± 0.025		303.38 ± 53.42	-
Okawa et al. (2021) [32]	cross-sectional study	Smokers	45	40/5	45	47.2 ± 6.3	0.646 ± 0.021	381.02 ± 40.31	382.0 ± 68.2	221.9 ± 238.6
		Nonsmokers	30	18/12	30	46.8 ± 5.5	0.653 ± 0.023	360.27 ± 127.92	339.3 ± 52.3	-

Note CVI, choroidal vascularity index; FRT, total retinal thickness; SFCT, subfoveal choroidal thickness; SD, standard deviation

**Table 2:** MINORS for assessing the quality of included studies

S.No	Methodological item for nonrandomized studies	Wei et al. (2019) [15]	Eski et al. (2023) [30]	Koçak et al. (2020) [31]	Okawa et al. (2021) [32]
1.	A clearly stated aim	2	2	2	2
2.	Inclusion of consecutive patients	2	1	2	2
3.	Prospective collection of data	0	0	2	0
4.	Endpoints appropriate to the aim of the study	2	2	2	2
5.	Unbiased assessment of the study endpoint	1	1	1	2
6.	Follow-up period appropriate to the aim of the study	2	2	2	2
7.	Loss to follow up less than 5%	2	2	2	2
8.	Prospective calculation of the study size	0	0	2	0
9.	An adequate control group	2	2	2	2
10.	Contemporary groups	2	2	2	2
11.	Baseline equivalence of groups	2	2	2	2
12.	Adequate statistical analyses	2	1	2	2
13.	MINORS score	19	17	23	20

MINORS, Methodological Index for Nonrandomized Studies

### Dose-response relationship between smoking intensity and choroidal vascularity index

**Fig. 7:**
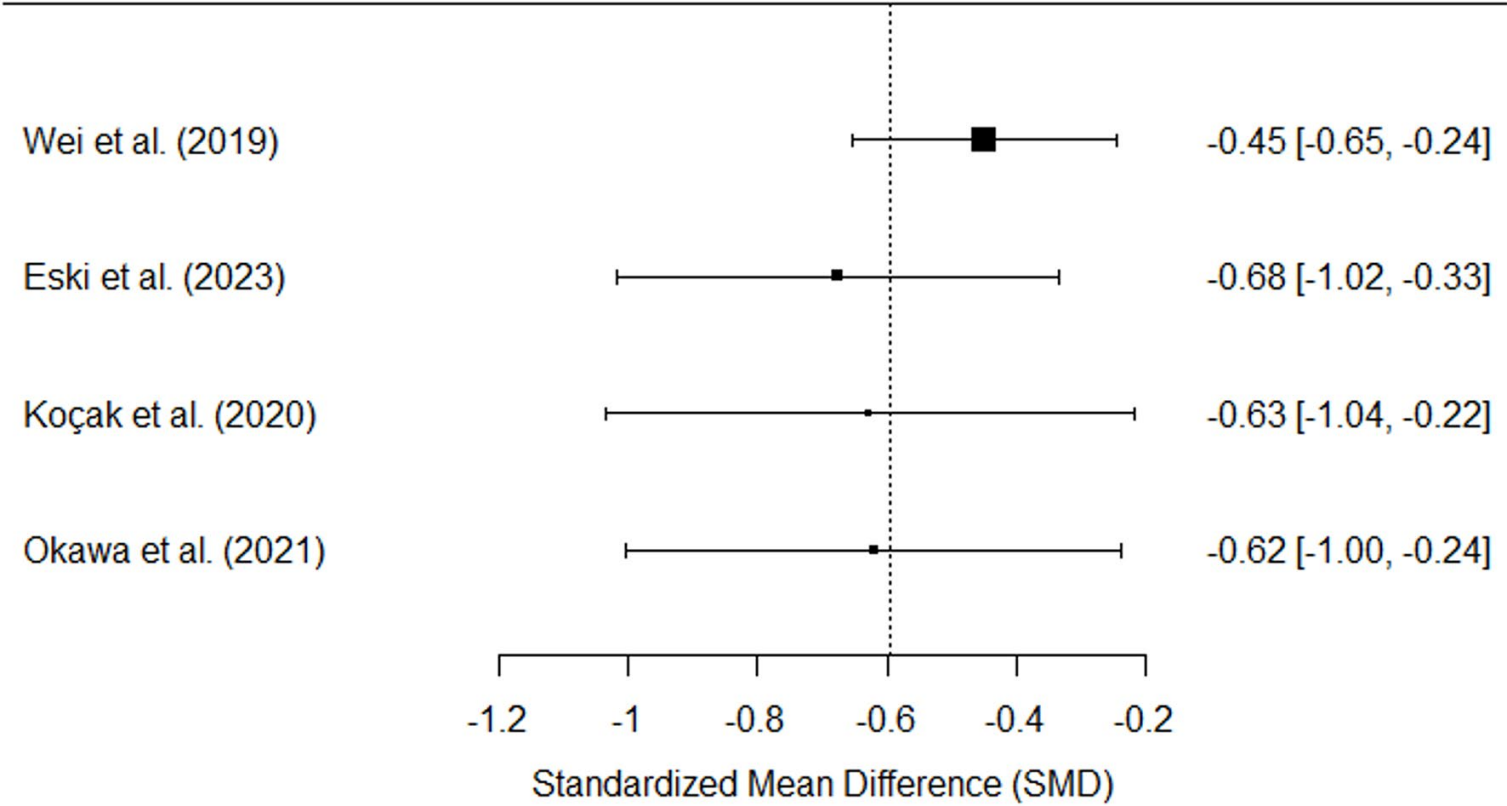
The dose-response relationship between pack-years and CVI shows a negative correlation (*r* = -0.78, *p* = 0.22), indicating a trend of decreased CVI with increased smoking intensity

This study performed a linear regression analysis to investigate the relationship between smoking intensity (measured in pack-years) and CVI. The results demonstrated a strong negative correlation (*r* = -0.78), suggesting that as smoking intensity increases, CVI tends to decrease. However, the association was not statistically significant (*p* = 0.22), likely due to the small sample size or variability in the data. Further studies with larger sample sizes are needed to confirm the dose-dependent impact of smoking on choroidal vascularity. (Fig. 7)

### Relationship between choroidal vascularity index and vision

We examined the relationship between CVI and BCVA to understand how smoking affects retinal health. After reviewing the studies in our analysis, we found that the BCVA data was reported inconsistently. This inconsistency limited our ability to conduct a thorough correlation analysis between CVI and BCVA. Only one study by Okawa et al. provided both CVI and BCVA data, which is insufficient for establishing a robust correlation across the entire dataset. Analyzing data from just one study will not yield meaningful results and will restrict the generalizability of our findings. Therefore, we cannot perform a comprehensive correlation analysis between CVI and BCVA.

## Discussion

This meta-analysis aimed to evaluate the impact of cigarette smoking on ocular health, specifically focusing on CVI, SFCT, and FRT, with a “leave-one-out” sensitivity analysis to enhance the robustness of the pooled effect size. Our comprehensive search identified 743 articles, of which only four studies, encompassing 702 eyes, met the inclusion criteria. The selected studies were diverse, including nonrandomized, retrospective, prospective, and cross-sectional. No evidence of publication bias was detected among the included studies.

The analysis revealed a significant reduction in the CVI among smokers compared to nonsmokers, with an SMD of -0.61 (95% CI: -0.78 to -0.43, *p* < 0.00001), indicating a strong negative impact or harmful effects of smoking on choroidal vascularity. Previous research has suggested that smoking triggers an immunological response in vascular and stromal tissues, leading to elevated levels of inflammatory markers [33]. According to recent studies [31, 34, 35], smoking may increase the stromal area by accumulating reactive inflammatory cells, causing leakage from dilated choroidal vessels and enlarging the luminal area through vasodilation due to thrombotic or secondary inflammatory responses. These findings supports the role of CVI in inflammatory ocular disorders. Therefore, the current study proposes and supports the hypothesis that CVI significantly decreases in smokers.

However, the effect of smoking on SFCT remains unclear. The overall mean difference of 3.88 (95% CI: -7.34 to 15.10) was not statistically significant, and substantial heterogeneity (I² = 79%) was present among the studies. This heterogeneity may be attributed to differences in study design (prospective vs. retrospective), smoking exposure classification (pack-years vs. current smoking status), and imaging methodologies used for CVI assessment. To further explore the impact of individual studies, we conducted a leave-one-out sensitivity analysis, systematically excluding one study at a time to assess its influence on the pooled effect size. The results showed that removing Wei et al. (2019) [15] had the most pronounced effect, shifting the pooled standardized mean difference (SMD) to -0.45 (95% CI: -0.65, -0.24) and reducing heterogeneity significantly. This suggests that Wei et al. (2019) [15] contributed to heterogeneity, potentially due to differences in participant demographics or OCT segmentation techniques. This finding aligns with a previously published meta-analysis by Yang et al. (2019) [29], which also reported no significant impact of smoking on CT.

However, subgroup analyses by region and study design have shown varying outcomes [29]. Moreover, Wei et al. (2019) [15] found no significant association between cigarette smoking and SFCT. The high heterogeneity observed in this study underscores the potential complexity of the effects of cigarette smoking on CT. The choroid, located between the retina and sclera, is rich in blood vessels and pigment cells. Smoking, which affects vascular endothelial cell function, can also affect the choroid [36]. However, CT is influenced by multiple factors, including systemic blood pressure, ocular parameters, and age-related changes. Therefore, further research is required to conclude the specific effects of smoking on CT confidently.. Notably, RPI has not been previously reported in the literature, indicating a gap in research on the vascular effects of smoking on retinal health. Our findings suggest that cigarette smoking adversely affects choroidal perfusion, as evidenced by decreased CVI, which correlates with decreased visual acuity. The hypothetical relationship proposed here indicates that cigarette smoking-induced vascular changes may impair macular microcirculation and ultimately affect vision. The current study examined the existing body of literature to uncover the potential implications of cigarette smoking on ocular abnormalities, offering valuable insights for both clinical practice and public health initiatives. The meta-analysis revealed a significant association between smoking and reduced CVI, with evidence suggesting a dose-response relationship. Smokers with a higher smoking burden showed more substantial reductions in CVI, indicating that smoking may have a cumulative detrimental effect on choroidal vascularity [30].

This meta-analysis showcases several strengths. First, a comprehensive literature search strategy ensured the inclusion of all available studies, enhancing the robustness of our findings. It encompassed all potential observational studies, allowing for thorough assessment and detailed analysis of the effects of smoking on CVI, SFCT, and FRT. Second, the in-depth comparative analyses provided more profound insights into the specific impact of tobacco on each parameter. Third, to our knowledge, this is the first study to review and analyze the literature on the effects of smoking on CVI and RPI, the latter of which has not been sufficiently reported to allow for its corresponding meta-analysis. A key strength of this study is the robustness of the findings, confirmed by multiple sensitivity analyses. The leave-one-out sensitivity analysis demonstrated that no single study disproportionately influenced the pooled effect size, affirming the consistency of the observed association between cigarette smoking and reduced CVI. Despite slight variations in effect size when individual studies were excluded, the negative association remained statistically significant throughout all scenarios, strengthening confidence in the meta-analysis conclusions. However, this meta-analysis also has several limitations. First, the variability in study designs (prospective, cross-sectional, or retrospective) may affect the generalizability of the conclusions. The inconsistency in the reported effects of smoking on retinal and choroidal thickness indicates that further validation through well-designed studies is necessary. Second, because the results came from observational studies, the evidence is inadequate to determine the impact of smoking on CVI definitively. Third, studies have not yet reported the effect of cigarette smoking on RPI, which has prevented us from drawing definitive conclusions in this area. Given the relatively small number of studies included, conducting advanced and well-designed research is crucial for better understanding these issues.

Additionally, the absence of longitudinal data restricts our understanding of the long-term effects of smoking on the choroidal microvasculature, highlighting the necessity for further research with extended follow-up periods. Finally, although our leave-one-out sensitivity analysis confirmed the stability of the pooled effect estimates, the high heterogeneity (I2 = 79%) indicates that variations in methodology at the study level may have influenced this variability. Given the significant heterogeneity, we recommend several strategies to reduce its impacts in future research. Subgroup analyses that categorize studies based on design, imaging modality, and smoking exposure classification may help pinpoint the underlying sources of variability. Furthermore, meta-regression can assess the effects of continuous variables, such as pack-years, age, and OCT imaging techniques, on pooled effect estimates. In addition, standardizing imaging methods, including OCT segmentation techniques and measurement protocols, would enhance comparability across studies and minimize inconsistencies in reported outcomes.

In conclusion, the trend of decreasing CVI mean values with increased smoking intensity strongly suggests a reduction in choroidal vascularity, and this relationship appears to be dose-dependent. Due to its noninvasive nature, CVI has the potential to serve as a predictive marker for systemic vascular dysfunction and the onset of ocular diseases, such as AMD, in smokers. Future research should explore the utility of CVI and RPI in this context to deepen our understanding of the broader vascular implications of smoking on other retinal parameters and potential confounding factors.

## Electronic supplementary material

Below is the link to the electronic supplementary material.

Supplementary Material 1

## Data Availability

No datasets were generated or analysed during the current study.

## Abbreviations

AMD: age-related macular degeneration
CFA: choriocapillaris flow area
CI: confidence interval
CT: Choroidal thickness
CVI: choroidal vascularity index
FRT: full-retinal thickness
LA: luminal area
MOOSE: Meta-analysis of Observational Studies in Epidemiology group
MINORS: Methodological Index for Nonrandomized Studies
NOS: Newcastle–Ottawa scale
OCT: Optical coherence tomography
OCTA: optical coherence tomography, angiography
PRISMA: Preferred Reporting Items for Systematic Reviews, and Meta-Analysis
RCTs: randomized controlled trials
RPI: retinal perfusion index
SD: standard deviation
SMD: standardized mean difference
SFCT: Subfoveal choroidal thickness
SA: stromal area
SE: standard error
VD: vessel density
