# Supplementary material for: Effects of cigarette smoking on retinal thickness and choroidal vascularity index: a systematic review and meta-analysis
Source: Int J Retina Vitreous. 2025 Feb 26;11:21. doi: 10.1186/s40942-025-00646-9 (PMC11866857; doi:10.1186/s40942-025-00646-9)
Supplement: Supplementary file 1 — Supplementary Material 1 [file 40942_2025_646_MOESM1_ESM.docx]

| **S.No** | **Keywords** | **Search Result** |
| --- | --- | --- |
| 1. | Smoking OR smoking OR Cigarette OR cigarette smoking OR cigarette consumption OR smoking behavior | 43,321 |
| 2. | Nicotine OR smoking cessation OR tobacco OR tobacco use disorder OR tobacco products | 17,702 |
| 3. | 1 OR 2 | 46,126 |
| 4. | Retinal Perfusion OR Retinal Perfusion Indices OR retinal perfusion indices **r**etinal blood flow OR retinal circulation OR retinal vascular density OR retinal vessel density OR retinal blood flow velocity OR retinal vascular autoregulation OR retinal artery diameter OR retinal vein diameter OR vessel index OR relative flow volume | 15,128 |
| 5. | Optical Coherence Tomography Angiography (OCTA) OR fluorescein angiography (FA) OR Optical Coherence Tomography Angiography (OCTA) OR Laser Speckle Flowgraphy OR Doppler Optical Coherence Tomography | 660 |
| 6. | 4 OR 5 | 15,399 |
| 7. | choroidal vascularity OR choroidal vascularity index OR CVI OR choroidal blood flow OR choroidal circulation OR choroidal thickness OR choroidal vascular density OR choroidal volume OR choroidal vessel diameter OR choroidal perfusion | 3,640 |
| 8. | Optical Coherence Tomography Angiography (OCTA) OR Enhanced Depth Imaging Optical Coherence Tomography (EDI-OCT) | 125 |
| 9. | 7 OR 8 | 3,666 |
| 10. | 3 AND 6 | 673 |
| 11. | 3 AND 9 | 35 |
| 12. | 10 AND 11 | 5 |
| 13 | 10 OR 11 | 703 |
|  |  |  |
|  |  |  |
|  |  |  |

**Supplementary information file**

**Effects of Cigarette Smoking on Retinal Perfusion Indices and Choroidal Vascularity Index: A systematic review and meta-analysis**
